# Longitudinal optical coherence tomography imaging of tissue repair and microvasculature regeneration and function after targeted cerebral ischemia

**DOI:** 10.1117/1.JBO.25.4.046002

**Published:** 2020-04-13

**Authors:** Yuankang Lu, Xuecong Lu, Cong Zhang, Paul J. Marchand, Frédéric Lesage

**Affiliations:** a Laboratoire d’Imagerie Optique et Moléculaire, École Polytechnique de Montréal, Montréal, Québec, Canada; b Université de Montreal, Montréal, Québec, Canada; c Institut de Cardiologie de Montréal, Montréal, Québec, Canada

**Keywords:** optical coherence tomography-angiography, photothrombosis, microvasculature, capillary stalling

## Abstract

**Significance:** Understanding how the brain recovers from cerebral tissue and vascular damage after an ischemic event can help develop new therapeutic strategies for the treatment of stroke.

**Aim:** We investigated cerebral tissue repair and microvasculature regeneration and function after a targeted ischemic stroke.

**Approach:** Following photothrombosis occlusion of microvasculature, chronic optical coherence tomography (OCT)-based angiography was used to track ischemic tissue repair and microvasculature regeneration at three different cortical depths and up to 28 days in awake animals. Capillary network orientation analysis was performed to study the structural pattern of newly formed microvasculature. Based on the time-resolved OCT-angiography, we also investigated capillary stalling, which is likely related to ischemic stroke-induced inflammation.

**Results:** Deeper cerebral tissue was found to have a larger ischemic area than shallower regions at any time point during the course of poststroke recovery, which suggests that cerebral tissue located deep in the cortex is more vulnerable. Regenerated microvasculature had a highly organized pattern at all cortical depths with a higher degree of structural reorganization in deeper regions. Additionally, capillary stalling event analysis revealed that cerebral ischemia augmented stalling events considerably.

**Conclusion:** Longitudinal OCT angiography reveals that regenerated capillary network has a highly directional pattern and an increased density and incidence of capillary stalling event.

## 1 Introduction

Stroke survivors are often left with serious, long-term disabilities, such as reduced mobility, declined cognition, and impaired sensation, due to permanent neurological damage to the brain. Evidence from animal models and human subjects both show that the brain has a certain degree of spontaneous plasticity after stroke,^1^^,^^2^ and a maximal functional recovery can be achieved in a time-limited plastic window by harnessing neuroplasticity.^3^^,^^4^ The poststroke neural network restoration was revealed to be tightly linked with angiogenesis.^5^ Newly formed vessels release signaling molecules to trigger the migration of newly born neurons into the ischemic region,^6^^–^^8^ suggesting a pivotal role of revascularization in stroke recovery.

A previous study using two-photon microscopy underlined that the orientation of the capillary network surrounding the lesion became highly organized after the ischemic event.^9^ However, this vascular rearrangement could not be thoroughly examined, since the fluorescent dye leaking through the damaged blood–brain barrier (BBB) considerably compromised the two-photon image quality. Optical coherence tomography (OCT), which provides a large field of view (FOV) and an extended imaging depth without the need for exogenous contrast agent, was also utilized to monitor the cerebral hemodynamic response and tissue property changes for the acute and chronic phases of stroke.^10^^,^^11^ However, they did not look into whether these responses to cerebral ischemia were depth-specific, despite studies showing that the oxygen environment is not homogeneous throughout the cortical depths.^12^^,^^13^ OCT-based angiography was also recently extended to detecting capillary stalling events,^14^ which are largely caused by leukocyte adhesion in capillary segments.^15^ Thus, OCT-angiography can be used as an indirect method to investigate poststroke inflammation in the newly formed microvessels.

A number of rodent stroke models have been developed to simulate human ischemic stroke in order to uncover the underlying physiological and molecular mechanisms and to test new therapeutic agents and rehabilitation strategies.^16^^–^^20^ In this study, we used a focal stroke model based on photothrombosis. We induced a targeted ischemic lesion in the mouse cortex. Poststroke follow-up was performed over 28 days using longitudinal OCT imaging. Since OCT does not require imaging contrast agent, the damaged BBB will not affect the image quality. The ischemic lesion size was characterized from OCT-angiograms, and tissue damage at different cortical depths was compared. A capillary orientation analysis was developed to quantify poststroke microvasculature reorganization. The progress of capillary network reorganization was tracked from the baseline till 28 days after the photothrombosis, and the correlation between microvasculature reorganization and depth was investigated. Capillary stalling events, assumed to be related to neutrophil adhesion,^15^ were analyzed for the baseline and the recovery phase (day 28) based on time-resolved OCT-angiograms.

## 2 Methods

### 2.1 Animal Preparation

The procedures and protocols were approved by the Animal Research Ethics Committee of the Montreal Heart Institute, and all animal experiments were performed in accordance with the Canadian Council on Animal Care recommendations. Six C57BL/6J male mice, between 3 and 6 months of age, were used in our study. A craniotomy was performed over the left barrel cortex (0.5-mm posterior to bregma and 3.5-mm lateral to the midline) to implant a chronic cranial window for OCT imaging. A region of skull with a diameter of 3 mm was removed using a microdrill after the scalp was retracted, and the dura was kept intact. The exposed brain surface was then covered with a stacked four-layer glass cover slip (3×3  mm and 1×5  mm diameter) and further sealed with dental acrylic cement to prevent infection and dehydration. A fixation bar was glued to the skull using the dental acrylic. Physiological parameters including electrocardiography, respiration, heart rate, and oxygen saturation of the isoflurane-anesthetized mouse were continuously monitored by a small animal physiological monitoring system (Labeo Technologies Inc., Canada), whose heated platform module also maintained the mouse body temperature at 37°C.

OCT measurements were taken in awake resting mice to avoid the modulation of vascular and neural physiology^21^^,^^22^ by anesthetics. During image recording sessions, the mice were placed on a free treadmill wheel with their head fixed on a metal frame by the surgically attached bar. Since OCT-angiography is phase-sensitive and that even subpixel motions will seriously affect the signal-to-noise ratio (SNR) of OCT-angiograms, it is important that the mice stay still during imaging sessions. Thus, training of the mice for head restraint prior to OCT measurements was required to habituate them to head fixation and reduce their stress. After a week of head-restraint training on the treadmill wheel, the mice were able to reach a resting state within 5 min after being fixed onto the setup. They were able to stay calm and still for periods of minutes separated by short bouts of locomotion. After the initial baseline measurement, the mice were still trained every day between imaging sessions to maintain their habituation to head restraint throughout the study. The mice were closely monitored for locomotion during image acquisitions.

### 2.2 Spectral-Domain OCT Imaging System

**Fig. 1:**
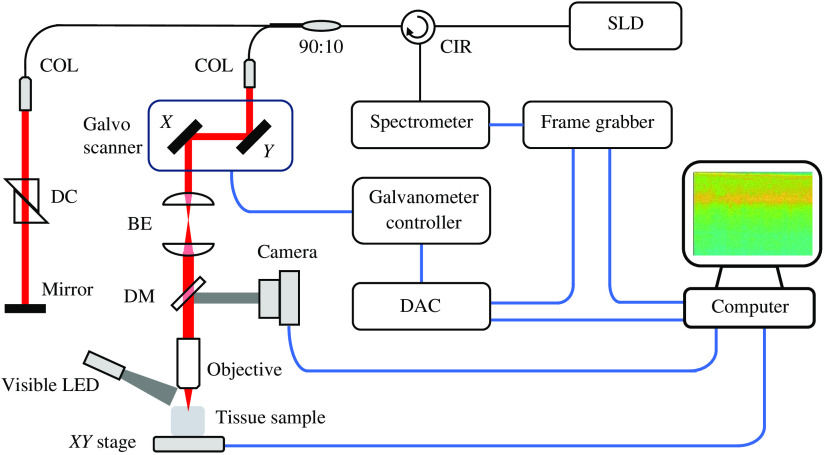
Schematic drawing of the OCT system. 1310-nm infrared light (the red beam) was emitted by an SLD and then split between the sample arm and the reference arm by a fiber optic coupler. The light beam in the sample arm was scanned across the FOV using a 2-D galvanometer system. The surface of the mouse cortex was illuminated by a second light source, a visible LED, for the purpose of wide-field imaging. The reflected visible light (the green beam) from the brain surface was deflected toward a CMOS camera by a dichroic mirror placed between the beam expander and the objective lens. The galvo scanner, the beam expander, the dichroic mirror, the camera, and the objective lens were assembled on an optical construction rail. The construction rail itself was mounted on the motorized vertical translation stage. For the sake of the clarity of the schematic, the construction rail and the vertical stage are not shown in the figure. SLD, superluminescent diode; CIR, circulator; 90:10, fiber optic coupler; COL, collimator; X-Y: XY 2-D galvo scanner; BE, beam expander; DM, dichroic mirror; DC, dispersion compensator; DAC, data acquisition card; LED, light-emitting diode.

Imaging of cortical structure and vasculature was performed with a home-build spectral-domain OCT. A broadband light source centered at 1310 nm from a superluminescent diode (SLD) (LS2000C, Thorlabs) was split between the sample arm and the reference arm by a 90:10 fiber optic coupler (TW1300R2A2, Thorlabs). A long working distance objective (M Plan Apo NIR 10X, Mitutoyo, Japan) was installed at the end of the sample arm to focus the collimated light beam into tissue sample. The spectral interferogram was registered by a spectrometer [Cobra 1300-(1235 to 1385 nm), Wasatch Photonics] and then digitized by a frame grabber (PCIe-1433, National Instruments). Dispersion mismatch between the two arms was first carefully compensated with N-SF11 compensation glass (Edmund Optics), and the small residual mismatch was then finely corrected with a numerical compensation technique.^23^ The axial resolution, defined as the full-width half-maximum, was measured to be 2 pixels on the tomogram, which is equivalent to about 8.3  μm in biological tissues, and its lateral resolution in tissue was about 2.3  μm. In the sample arm, a dichroic filter was placed to transmit the infrared light used by OCT system and deflect the visible light for wide-field imaging. The wide-field imaging helped locate the region of interest (ROI) to be scanned by OCT. A detailed schematic drawing of the imaging system is shown in Fig. 1.

The sample arm (consisting of a galvanometer scanner, a beam expander, and an objective lens) was mounted on a motorized vertical translation stage (MLJ150/M, Thorlabs). The objective lens can thus be elevated or lowered by the vertical stage in order to adjust the depth of the imaging focal point in the mouse cortex. The treadmill wheel onto which the mouse was attached was fixed on a motorized XY linear translation stage (T-LSR, Zaber Technologies, Canada) for fine adjustment of the relative lateral position of the cranial window with respect to the light beam. The three-axis motion control was integrated into our acquisition software.

### 2.3 Localized Photothrombosis of Cerebral Microvasculature

The ischemic stroke model was created using a photochemical reaction introduced by Watson.^24^ Mice were first intraperitoneally administered Rose Bengal (15  mg/ml, 0.2 ml), a photosensitive dye. A selected cortical region free of large vessels was irradiated by a focused green laser beam, since large-vessel thrombosis could lead to a less predictable and less controlled outcome. In addition, avoiding regions with large pial vessels could also minimize the effect of tail artifacts in OCT angiography images.^25^ Under green light illumination, free radicals produced by Rose Bengal damage the endothelium of the microvasculature in the targeted region, which thereby triggers discoid platelet aggregation and leads to microvascular thrombotic occlusions. As the microvasculature plays a critical role in cerebral oxygen supply and blood flow regulation,^26^^–^^28^ the surrounding brain parenchyma of the clotted capillaries suffers from ischemia and even infarction. In this way, precise induction of a localized thrombotic lesion in the mouse cortex was achieved. The whole process of photothrombosis was monitored using a home-built laser speckle imaging system. This laser speckle imaging system has an FOV of 2.6  mm×2.2  mm with a resolution of 7  μm.

### 2.4 Optical Coherence Tomography Angiography

A 1  mm×1  mm ROI was chosen with the photothrombosis-induced ischemic lesion located in the center. Volumetric scans of the cortex contained 450 B-frames, each of which was composed of 500 A-lines. Raw spectra were first resampled in k-space and then multiplied by a Hanning window before inverse Fourier transform was applied to obtain three-dimensional (3-D) complex-valued OCT structural images. B-scans were repeated twice at each position along the slow axis for angiography imaging. Global phase fluctuations (GPF) caused by subpixel motion within repeated B-frames were corrected based on the assumption that dynamic tissue only accounts for a very small percentage of brain tissue and that phase and intensity of light reflected from static tissue remain constant.^29^ Since light reflected by moving red blood cells (RBCs) experiences a large phase shift and/or a big intensity change, the vessel network can be extracted based on the phase and intensity difference between GPF-corrected repeated B-frames.^30^ The resulting 3-D angiograms were filtered with a 3-D Gaussian smoothing kernel with a standard deviation equal to 1 pixel in all three dimensions. A fast axis scan rate of 90 Hz was used, which resulted in an acquisition time of 10 s per volumetric scan. The ROI was scanned repeatedly 100 times for 16.7 min to get a series of OCT-angiograms. As short bouts of animal locomotion were inevitable during image acquisition, motion-artifact-corrupted volumes were removed manually, after visual inspection, and out of the entire acquisition, only the first 60 volumetric angiograms free of motion artifacts were kept for further analysis for each mouse.

In order to extract information of the capillary network at multiple depths in the cortex, three time-resolved OCT-angiographies were performed in the same ROI with light beam being focused at three different cortical depths, namely 250, 400, and 550  μm beneath the cortical surface. The shift of the axial focus position in the brain tissue was achieved by the vertical translation of the objective lens in the sample arm mounted on the vertical translation stage.

The chronic study performed six OCT imaging sessions over 28 days. The baseline measurement was taken 1 day before photothrombosis. The second imaging session took place 3 days after the thrombotic lesion was induced in the mouse brain, and the following four measurements were made 8, 14, 21, and 28 days, respectively, after the ischemic stroke event.

### 2.5 Capillary Orientation Analysis

**Fig. 2:**
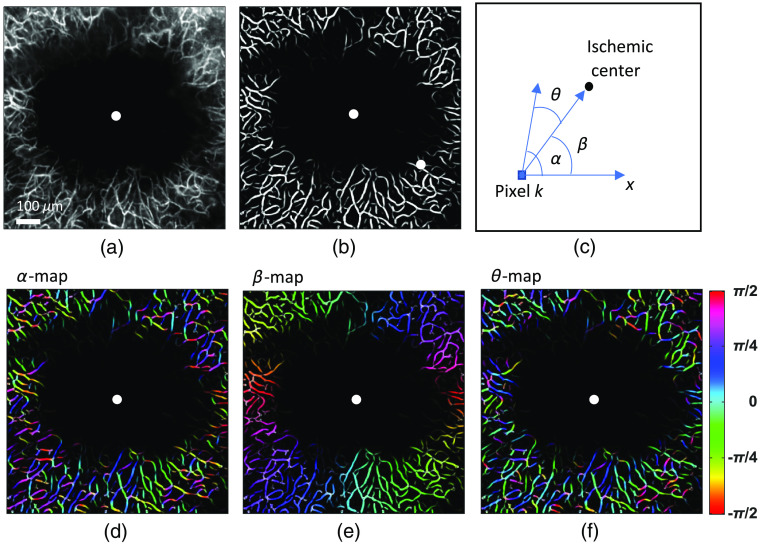
Computation of capillary angular orientation map. (a) MIP of an angiogram (490 to 610 μm under the cortical surface) acquired from an example mouse 21 days after the photothrombosis. (b) Tubeness-filter and Frangi-filter enhanced angiogram. (c) The local orientation (α) of each pixel with respect to x axis can be assessed using OrientationJ, a plugin running on ImageJ. The direction (β) of the vector pointing from a given pixel toward the ischemic center can be obtained as well. The local orientation (θ) of each pixel relative to the direction of the ischemic center can thus be estimated by the difference between α and β. (d) The α-orientation map, (e) the β-orientation map, and (f) the θ-orientation map, where the angle value (α,β, or θ) is encoded in color. The θ-orientation map will be further utilized to analyze the local orientation distribution of capillary segments. The white dot in (a), (b), (d), (e), and (f) indicates the estimated ischemic center. (d), (e), and (f) Share the same colorbar.

The morphology of cerebral microvascular network and its hemodynamic function are tightly related.^31^^,^^32^ In our study, an image processing strategy was established to analyze the orientation of capillary segments. A preprocessing step of averaging 60 volumetric angiograms was taken to increase the SNR before images were further processed and analyzed. The 3-D angiogram was then flattened into an *en face* 2-D plane along the z direction around the focal plane (±15  pixels) using the maximum intensity projection (MIP) [Fig. 2(a)]. A tubeness filter^33^ and a Frangi filter^34^ were sequentially applied to the MIP image to enhance the vessel structure and reduce the background signal from the parenchyma [Fig. 2(b)]. Both filters are implemented in ImageJ (NIH, Maryland). Local orientation and coherence were computed for each pixel of the filter-enhanced 2-D *en face* angiogram based on its structure tensor using OrientationJ,^35^ an ImageJ plug-in. The coherence value with a range between 0 and 1 indicates the degree of local isotropy. Highly oriented structures such as capillaries tend to have high values on the coherence map, while isotropic regions such as ischemic zones usually possess low coherence values. The output image of OrientationJ is encoded in HSV (hue, saturation, and value) color space, with each of the three dimensions representing the orientation, coherence, and input image brightness, respectively [Fig. 2(d)]. The local orientation of each pixel given by OrientationJ is relative to the X axis [denoted by α in Fig. 2(c)], and the orientation to be quantified is with respect to the direction defined by the pixel itself and the center of the ischemic lesion [denoted by θ in Fig. 2(c)]. In order to remove this pixel-specific angle offset [denoted by β in Fig. 2(c)], an orientation map was synthesized based on the relative position between each pixel and the infarct center [Fig. 2(e)]. By subtracting the synthesized orientation map from the original one obtained with OrientationJ, a final orientation map can be restored with the hue value of each pixel being the angular orientation with respect to the vector pointing from the given pixel toward the ischemic center [Fig. 2(f)]. A binary mask for the capillary network can be generated by applying a threshold to the vessel-enhanced 2-D angiogram. This mask was then multiplied with the final orientation map so that parenchymal tissue could be suppressed, and only capillary segments were included for the angular distribution analysis. Capillary segments with angular orientation of between ±15  deg (∼±0.26  rad) were considered to be aligned with the direction of the ischemic center.

### 2.6 Capillary Stalling Event Analysis

**Fig. 3:**
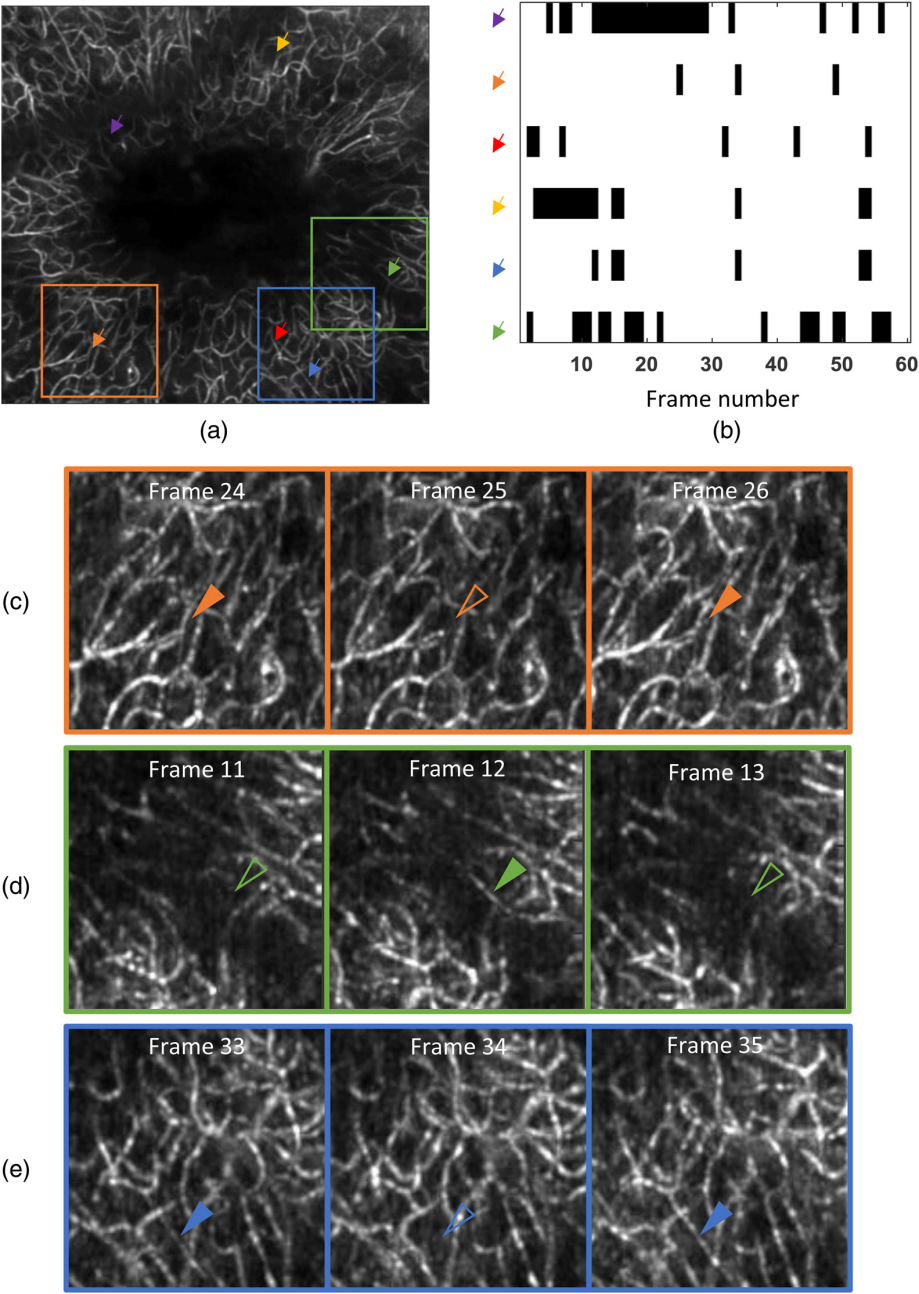
Identification of stalled capillary segments from OCT-angiogram time series. (a) *En face* MIP of a 3-D angiogram acquired in an example mouse 28 days after the photothrombosis. Six of the capillaries experiencing stalling events are indicated by arrows of different colors. (b) Stalling event timeline throughout 60 frames for the six capillary segments, where stalling events are labeled as black spots. Each arrow represents one capillary segment, and their color matches with that of the arrows used in (a). (c)–(e) Zoomed-in ROI taken from (a). The capillaries undergoing stalling events are marked by arrowhead. Solid ones are used to indicate the moments when the capillary segments could be seen, and hollow ones are used to indicate the moments when the capillaries disappeared, namely that they were stalled.

The stalling event analysis was based on the time series of 60 OCT-angiograms, which were acquired sequentially in time. However, it should be noted that the 60 volumes were not necessarily evenly spaced in time. The animal’s resting state was interrupted by short bouts of locomotion (lasting 3 to 10 frames), these motion-artifact-corrupted volumes were discarded, and the first 60 angiograms free of motion artifacts were used for stalling analysis. The volumetric angiograms within the depth of field, where capillaries were well resolved, were flattened into an *en face* 2-D angiogram using MIP for the stalling event quantification. Stalled capillary segments were identified visually and stalling events were counted. When a capillary segment stalls, its intensity on the OCT-angiogram drops dramatically to the level of the background. The stalling event was thus defined as the complete disappearance of a whole capillary segment on the angiograms [Figs. 3(c)–3(e)]. Since the time interval between two consecutive volumes (in the 60-volume dataset) is large (≥10  s), which is determined by our imaging protocol, the detection of a stalled capillary segment on one frame only means that the RBC flow in that segment is interrupted at the very moment when the capillary is being scanned and can hardly lead to the conclusion that the capillary segment is stalled for the whole 10 s during which the whole volume is being scanned. Thus, we use the term “stalling event counts.” The stalling event density and stall incidence were investigated at three cortical depths for each mouse. The first parameter, stalling event density was calculated by the total number of identified stalling events on the 60 angiograms divided by the area of the vascularized region. Since very few capillaries were seen inside the ischemic lesion, this region was excluded for the calculation of the stalling event density. The stall incidence was defined as the ratio of the number of stalled capillary segments to the total number of capillary segments in the ROI.

## 3 Results

### 3.1 Deeper Cerebral Tissue Is More Affected by Photothrombosis

**Fig. 4:**
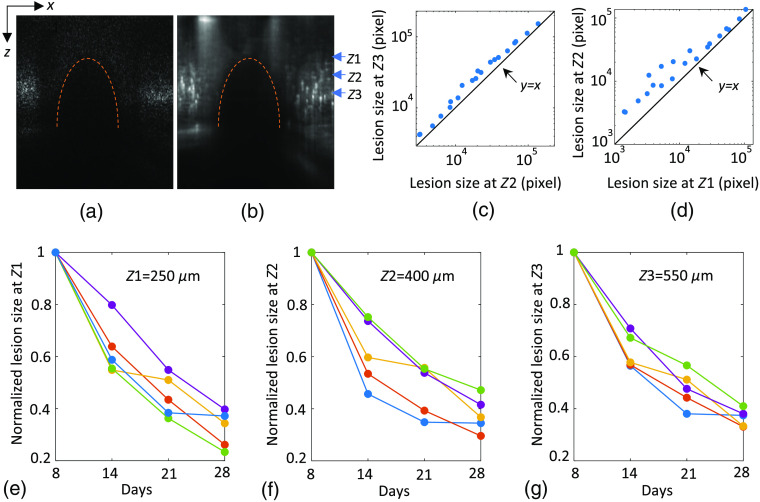
(a) Cross-sectional structural image running through the middle of the ischemic lesion. The ischemic region delimited by the orange dashed arc shows much lower intensity compared with the surrounding normal tissue. (b) Cross-sectional angiographic image at the same lateral position as (a). No capillaries are observed inside the ischemic lesion (marked by the orange dashed arc), where the intensity is also significantly lower than the surrounding tissue. Z1 (=250  μm), Z2 (=400  μm), and Z3 (550  μm) are the axial positions where the lesion area was measured. (c) Scatter chart showing the area of ischemic lesions at Z2 and Z3. (d) Scatter plot displaying the area of ischemic lesions at Z1 and Z2. Each dot corresponds to a measurement along the 28 days. The values are expressed in pixels. All the dots are located above the line y=x in both (c) and (d). (e)–(g) Case-by-case plot of normalized lesion size at the depth of 250, 400, and 550  μm from five mice over 28 days following the photothrombosis. The lesion size at each depth for each animal is normalized by its value of day 8.

**Fig. 5:**
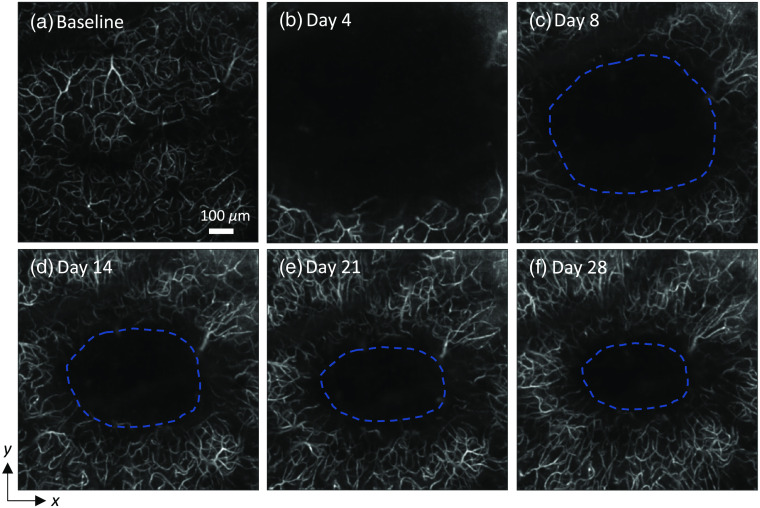
Postphotothrombosis microvasculature regeneration and cerebral tissue recovery. (a)–(f) A series of *en face* MIP of 3-D angiograms (400  μm beneath the cortical surface) acquired in an example mouse over the course of 28 days following the photothrombosis. Baseline data were acquired before the photothrombosis. Five follow-up OCT imaging sessions took place 4, 8, 14, 21, and 28 days after the photothrombosis. The ischemic lesion is delimited by a blue dashed line. All images share the scale bar.

To investigate the impact of photothrombosis on brain tissue, lesion area was quantified at different cortical depths using OCT imaging. The ischemic region showed a significant lower intensity than the surrounding normal tissue on the structural OCT image [Fig. 4(a)], suggesting that the tissue damage from cerebral ischemia led to a higher attenuation coefficient. The angiogram further enhanced the contrast between the ischemic lesion and its surrounding tissue [Fig. 4(b)]. The cross-sectional images [Figs. 4(a) and 4(b)] showed that the ischemic region started from the depth of around 200  μm under the cortical surface and stretched down to the white matter. The lesion contour [indicated by an orange dashed line in Figs. 4(a) and 4(b)] had a semielliptical form. The transverse area of the lesion can be estimated from the MIP of the angiograms as shown in Fig. 5. The ischemic region was not vascularized in contrast to the surrounding tissue. The segmentation of the lesion area on the 2-D angiogram was performed manually in the software ImageJ. The lesion size was quantified at three different depths, 250, 400, and 550  μm beneath the brain surface. Since the lesion area was still large on day 4 and the ROI did not cover the entire lesion in some cases [as shown in Fig. 5(b)], the evaluation of lesion size was performed starting from day 8 (when the ischemic area could be accurately measured). Case-by-case plots of lesion area evolution over time at the three aforementioned depths for five mice are shown in Figs. 4(e) and 4(g). The area of ischemic lesions diminished considerably over the course of 28 days compared with their initial size at all three depths for all mice. The lesion area was also compared between the depth of 400 and 250  μm [Fig. 4(c)] and between the depth of 550 and 400  μm [Fig. 4(d)]. It can be seen in Fig. 4(c) that all the points are above the line defined by y=x, indicating that the lesion area at the depth of 400  μm was larger than that at the depth of 250  μm. The same observation can be made in Fig. 4(d) as well, meaning that ischemic lesions had a bigger area at the depth of 550  μm than at the depth of 400  μm. These two scatter plots show that the ischemic lesion area increased with depth.

### 3.2 Photothrombosis-Induced Ischemic Lesion Causes Microvasculature Reorganization

**Fig. 6:**
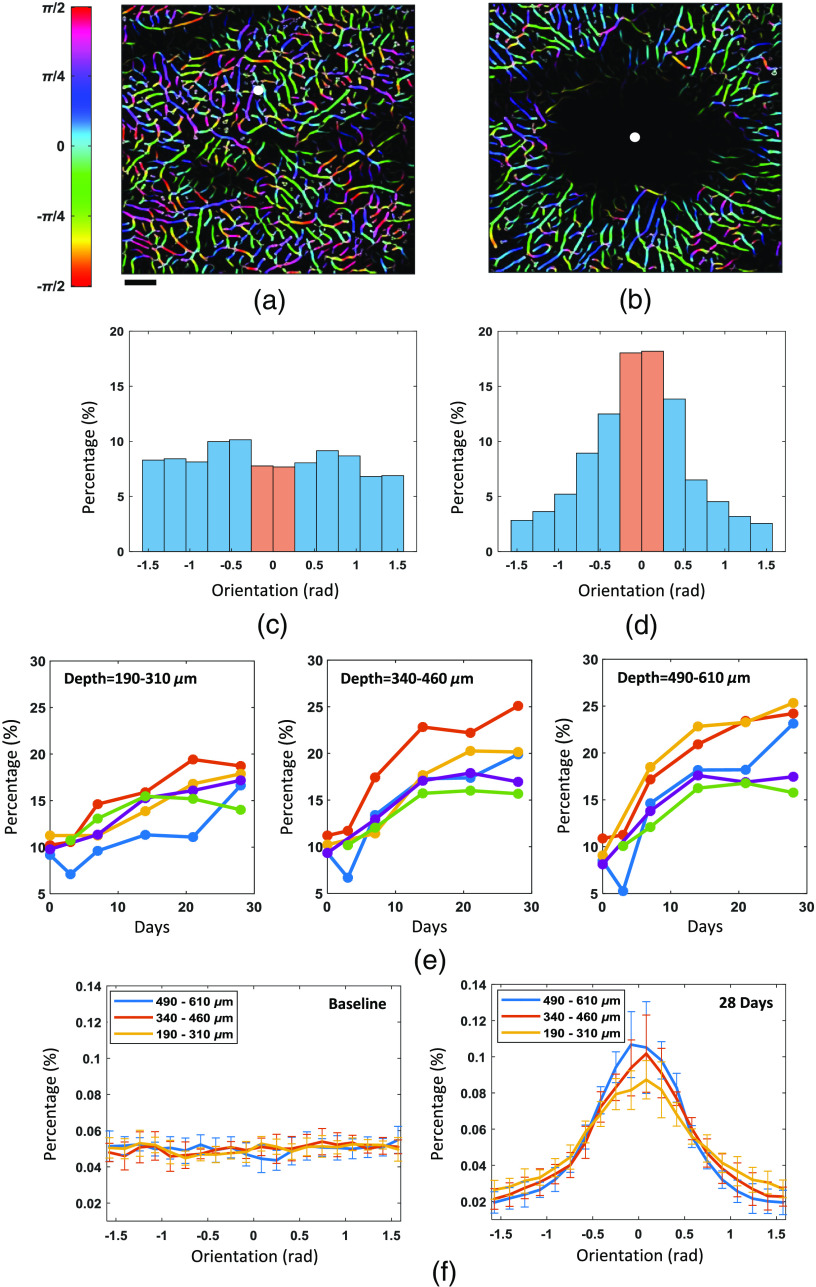
Visualization and quantification of capillary orientation. (a), (b) Tubeness-filter and Frangi-filter-enhanced MIP of capillary vasculature (at the depth of between 490 and 610  μm) acquired from an example mouse before and 28 days after the photothrombosis. The flattened angiograms are hue–saturation–brightness (HSB) color-coded with hue representing the capillary local orientation with respect to the direction pointing toward the white dot which coincides with the ischemic center. The images (a) and (b) are slightly shifted. The scale bar=0.1  mm. Panels (a) and (b) share the same scale bar and colorbar. (c) Distribution of capillary orientation from (a). The capillary orientation is uniformly distributed between −π/2 and π/2. (d) The distribution of capillary orientation derived from (b) displays a strong peak around 0 rad. The orange bins in the histograms (c) and (d) represent capillary segments whose orientation with respect to the ischemic center is within [−π/12,π/12], and these capillary segments are considered to be aligned with the direction of the ischemic center. (e) Case-by-case plots of the evolution of the percentage of the capillary segments which were aligned with the direction of the ischemic center at three different depth ranges for the five mice during the course of 28 days. (f) Distribution of capillary angular orientation before and 28 days after the photothrombosis at the three aforementioned depths ranges. The data in (f) are presented as mean±standard deviation (STD).

The cerebral microvascular network is organized in a way that optimizes oxygen delivery to brain tissue.^36^ The ischemic lesion in a great need of oxygen supply had a visible impact on the surrounding capillary network organization, as can be seen from the series of 2-D angiograms over time (Fig. 5): regenerated capillaries tended to grow toward the ischemic region. This change in microvasculature organization can be quantified using the capillary orientation analysis strategy detailed in Sec. 2. An example of the quantification of microvasculature organization is presented in Figs. 6(a)–6(d). The postprocessed angiograms had an extra dimension, the color, which indicated the local angular orientation of the capillary segments. No predominant orientation/color was observed in this baseline angiogram [Fig. 6(a)], while in the angiogram of day 28, the color of the microvasculature mostly lay in the spectrum between green and blue [Fig. 6(b)]. The corresponding angular orientation distributions of the two example angiograms were then computed. The orientation of the capillary segments on the baseline angiogram was uniformly distributed over the interval [−π/2,π/2] [Fig. 6(c)]. Conversely, the distribution based on the angiogram of day 28 was instead concentrated around 0 rad [Fig. 6(d)]: the percentage of the capillary segments perpendicular to the direction of the ischemic center dropped by more than half, and the percentage of the capillary segments pointing toward the lesion center increased to twice its original value. The progression of microvasculature reorganization, which was quantified by the percentage of the capillary segments aligned with the direction of ischemic center (between ±15  deg), was tracked at three different depths (250, 400, and 550  μm beneath the cortical surface) in the cortex of five mice. The case-by-case plots show that the microvasculature reorganization progressed steadily over time at all three cortical depths [Fig. 6(e)]. Furthermore, the degree of capillary reorganization was associated with the cortical depth. As data from day 28 show, the group-averaged capillary orientation distribution in the deepest layer had the highest peak and the distribution at the shallowest layer had the lowest peak [Fig. 6(f)].

### 3.3 Increased Capillary Stalling Events in Reorganized Capillaries

**Fig. 7:**
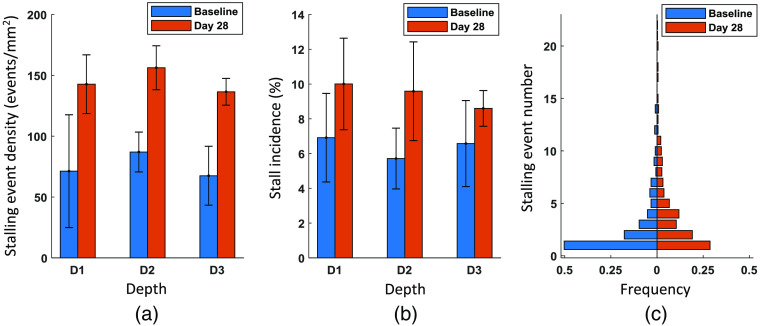
Comparison of capillary stalling events between the baseline and the measurement taken 28 days after photothrombosis. (a) Stalling event density and (b) stall incidence measured from baseline and day-28 OCT-angiogram time series at three different depths beneath the cortical surface. D1, D2, and D3 represent the depth ranges 230 to 270  μm, 380 to 420  μm, and 530 to 570  μm. The data are presented as mean±STD. (c) Distribution of stalled capillary segments in terms of the stalling event number for the baseline measurement and day-28 measurement.

Capillary stalling occurs when RBC flux is obstructed in capillary segments, which has been reported to be potentially caused by neutrophil adhesion.^15^ We analyzed capillary stalling events before and after photothrombosis in *de novo* vasculature. Baseline data from one mouse were corrupted and not used in this analysis. Since OCT-angiography is based on signals scattered by moving particles, stalled RBCs make capillaries disappear on OCT-angiograms. Capillary stalling events were thus detected by identifying the transition between the appearance and disappearance of capillary segments based on the time series of OCT-angiograms [Figs. 3(c) and 3(d)]. Figure 3(a) shows an example angiogram on which some of the stalled capillaries are indicated. The stalling event timeline of these capillaries throughout 60 frames was drawn [Fig. 3(b)]. Stalling events and stalled capillary segments were counted on the time series of OCT-angiograms (MIP) acquired at the three aforementioned cortical depths for the baseline and for day 28. Stalling event density, a measurement of stalling event number per unit area, approximately doubled at all three depths after the photothrombosis [Fig. 7(a)]. Stall incidence also increased considerably at all the three depths on day 28 compared with the baseline [Fig. 7(b)], indicating that new capillaries following ischemic injury were more likely to be stalled. Neither of these two parameters had a clear trend with the cortical depth neither before nor 28 days after the photothrombosis. The distribution of stalled capillary segments in terms of stalling event number was calculated for both the baseline and day 28 [Fig. 7(c)]. Before the photothrombosis, almost half of the stalled capillaries had ceased RBC flow only once. However, this proportion dropped to nearly a quarter on day 28. Namely, repeated stalling events in the same capillary segments happened more often after the photothrombosis than the baseline.

## 4 Discussion

In this work, we used longitudinal OCT imaging to study tissue recovery and microvasculature regeneration after cerebral ischemia in awake, resting mice. Cerebral tissues in deeper cortical layers were found to have larger ischemic lesions. Newly generated capillaries in the ischemic penumbra were highly directional, which could ensure an adequate blood supply to the ischemic lesion. Moreover, capillary orientation analysis revealed that microvasculature at deeper cortical depth displayed a more highly organized structure. We also unveil that induction of ischemic lesion in the mouse cortex substantially increased the frequency of capillary stalling events and the incidence of stalled capillaries.

During the photothrombosis procedure, the green laser was focused at the surface of mouse cortex. According to the Beer–Lambert law, light intensity decreases exponentially along the depth direction. Thus, the cortical layer close to the surface was exposed to the largest amount of photons, and capillaries near the surface should be more severely damaged than that in deeper layers. However, cross-sectional OCT structural and angiographic images showed that ischemic lesions only started from the depth of around 200  μm, potentially due to the lack of significant capillaries prior to this depth. Furthermore, the lesion size has a positive correlation with the depth. The result suggests that cerebral tissue at different cortical depths does not have the same resistance to ischemia induced by photothrombosis: deep tissue is more prone to getting damaged from ischemic stroke than superficial tissue. This finding could be due to the fact that superficial tissue in the cortex is not as densely vascularized as deep tissue,^37^ and the oxygen supply of superficial tissue largely relies on arterioles instead of capillary bed.^38^ As a result, the photothrombosis, which mainly damaged capillaries in our case, affected parenchymal tissue more seriously in deeper cortical layers. However, the effect of the divergence of the laser beam used in the photothrombosis is yet to be determined.

Revascularization after ischemic stroke plays an essential role in cerebral tissue repair.^39^ The neovasculature in the ischemic penumbra is thus expected to be organized in a way to maximize the tissue repair. A previous longitudinal two-photon study on a mouse stroke model discovered that the newly formed capillary segments tended to grow toward the ischemic zone and the regenerated microvasculature became more aligned with the direction of ischemic center over the course of stroke recovery.^9^ However, the biggest disadvantage of two-photon microscopy for investigating the poststroke microvasculature is that the fluorescent dye leaks through the damaged BBB and corrupts the angiogram. Since OCT-angiography does not need any exogenous contrast agent, imaging sessions can start from a very early phase of vascular recovery and the whole microvasculature can be imaged for further analysis. In this work, we also established a new strategy for capillary orientation analysis. The local angular orientation of capillary segments can be directly visualized on the color-encoded angiogram, and the distribution of capillary angular orientation can be accurately analyzed by including all capillary segments. We explored the microvasculature rearrangement at three different cortical depths from 250 to 550 μm. Over the course of 28 days after the photothrombosis, we observed a similar trend of capillary network reorganization to that reported in the aforementioned two-photon study. Regenerated capillaries stretched toward the ischemic region while the area of ischemic lesion diminished gradually. Multiple studies demonstrated that poststroke neovascularization was responsible for triggering the migration of neuroblasts and blood vessels were served as physical scaffolds for neural migration.^7^^,^^8^^,^^40^ This highly organized structural pattern of microvasculature in the ischemic penumbra can both ensure a more efficient oxygen supply to the ischemic region and provide a more directional migration route for neuroblasts. The molecular mechanism driving the microvasculature reorganization has yet to be elucidated. We found that although capillary network at all depths had a similar trend of reorganization, the deeper microvasculature was better aligned with the direction of the ischemic center than the superficial one. This finding is coherent with our earlier observation of ischemic lesion area: a deeper location corresponded with a larger ischemic region. This might be explained by the fact that oxygen supply in deep cerebral tissue completely relies on capillary bed, while for brain tissue close to the surface, the decline of oxygen supply from capillaries can be partially compensated by diving arterioles.^38^

Capillary stalling, seen as a sudden RBC obstruction in perfused capillaries, was shown to contribute to the reduction of local cerebral blood flow in an Alzheimer’s disease mouse model.^15^ The plugging of individual capillary segments was reported to be largely due to the adhesion of neutrophils in capillary segments.^15^ Ischemic stroke triggers rapid neutrophil response, and an increased number of neutrophils can be found in the brain after cerebra ischemia.^41^^,^^42^ In our study, we compared capillary stalling events between the baseline and day 28 after photothrombosis. Our data showed that a much higher number of stalling events per unit area were identified in the cortex 28 days after stroke than the baseline. Besides, the stalled capillary incidence in the poststroke recovery phase also increased considerably compared with the baseline. The increased stalling events and stalled capillary segments in the surrounding area of ischemic region may reduce the local cerebral flood flow as in the case of Alzheimer’s disease. However, though the chance is high, we cannot conclude that the increased capillary segment obstruction we observed in our study was caused by a higher level of neutrophils following ischemic stroke. Additional measurements will be needed to support this assumption. In our work, capillary stalling events were identified and manually counted as described in previous literature,^14^ which was time-consuming and arduous. Future work will therefore be dedicated to developing automatic approaches, which could ultimately help increase the efficiency of stalling event detection. Future work should also be focused on the nature of the poststroke capillary stalling. Finding new ways to reduce capillary stalling after ischemic stroke could be useful in establishing new therapeutic strategies for stroke treatment.

## 5 Conclusion

In this study, we used longitudinal OCT imaging to investigate the poststroke cerebral parenchymal tissue repair and microvasculature regeneration and function. OCT-angiography provides a good contrast to separate ischemic lesion from unaffected cerebral tissue. Tissues located deeper in the cortex are more vulnerable to cerebral ischemia than superficial ones. Cerebral tissue repair is accompanied by microvascular regeneration in the ischemic penumbra. Additionally, the newly generated capillary network has a highly directional pattern, which might optimize the blood perfusion in the ischemic zone and guide newly born neurons toward the lesion. The degree of microvasculature reorganization is also depth-dependant, as is the ischemic lesion size: deeper capillaries are more highly oriented than superficial ones. Ischemic stroke increases stalling event density and stalled capillary incidence, which is possibly caused by leukocyte binding to the capillary wall.

## References

[1] Harrison T. C. et al., “Displacement of sensory maps and disorganization of motor cortex after targeted stroke in mice,” Stroke 44(8), 2300–2306 (2013). SJCCA7 0039-2499 10.1161/STROKEAHA.113.001272 23743973

[2] Cicinelli P. et al., “Interhemispheric asymmetries of motor cortex excitability in the postacute stroke stage,” Stroke 34(11), 2653–2658 (2003). SJCCA7 0039-2499 10.1161/01.STR.0000092122.96722.72 14551397

[3] Murphy T. H. Corbett D., “Plasticity during stroke recovery: from synapse to behaviour,” Nat. Rev. Neurosci. 10(12), 861–872 (2009). NRNAAN 1471-003X 10.1038/nrn2735 19888284

[4] Hara Y., “Brain plasticity and rehabilitation in stroke patients,” J. Nippon Med. Sch. 82(1), 4–13 (2015). 10.1272/jnms.82.4 25797869

[5] Font M. A. Arboix A. Krupinski J., “Angiogenesis, neurogenesis and neuroplasticity in ischemic stroke,” Curr. Cardiol. Rev. 6(3), 238–244 (2010). 10.2174/157340310791658802 21804783 PMC2994116

[6] Carmichael S. T., “Cellular and molecular mechanisms of neural repair after stroke: making waves,” Ann. Neurol. 59(5), 735–742 (2006). 10.1002/ana.20845 16634041

[r7] Thored P. et al., “Long-term neuroblast migration along blood vessels in an area with transient angiogenesis and increased vascularization after stroke,” Stroke 38(11), 3032–3039 (2007). SJCCA7 0039-2499 10.1161/STROKEAHA.107.488445 17901386

[8] Ohab J. J. et al., “A neurovascular niche for neurogenesis after stroke,” J. Neurosci. 26(50), 13007–13016 (2006). JNRSDS 0270-6474 10.1523/JNEUROSCI.4323-06.2006 17167090 PMC6674957

[9] Schrandt C. J. et al., “Chronic monitoring of vascular progression after ischemic stroke using multiexposure speckle imaging and two-photon fluorescence microscopy,” J. Cereb. Blood Flow Metab. 35(6), 933–942 (2015). 10.1038/jcbfm.2015.26 25712498 PMC4640252

[10] Choi W. J. Li Y. Wang R. K., “Monitoring acute stroke progression: multi-parametric OCT imaging of cortical perfusion, flow, and tissue scattering in a mouse model of permanent focal ischemia,” IEEE Trans. Med. Imaging 38(6), 1427–1437 (2019). ITMID4 0278-0062 10.1109/TMI.2019.2895779 30714910 PMC6660833

[11] Srinivasan V. J. et al., “Multiparametric, longitudinal optical coherence tomography imaging reveals acute injury and chronic recovery in experimental ischemic stroke,” PLoS One 8(8), e71478 (2013). POLNCL 1932-6203 10.1371/journal.pone.0071478 23940761 PMC3737090

[12] Sakadžić S. et al., “Two-photon high-resolution measurement of partial pressure of oxygen in cerebral vasculature and tissue,” Nat. Methods 7(9), 755–759 (2010).1548-7091 10.1038/nmeth.1490 20693997 PMC2932799

[13] Devor A. et al., “‘Overshoot’ of O_2_ is required to maintain baseline tissue oxygenation at locations distal to blood vessels,” J. Neurosci. 31(38), 13676–13681 (2011). JNRSDS 0270-6474 10.1523/JNEUROSCI.1968-11.2011 21940458 PMC3188944

[14] Erdener Ş. E. et al., “Spatio-temporal dynamics of cerebral capillary segments with stalling red blood cells,” J. Cereb. Blood Flow Metab. 39(5), 886–900 (2019). 10.1177/0271678X17743877 29168661 PMC6501506

[15] Hernández J. C. C. et al., “Neutrophil adhesion in brain capillaries reduces cortical blood flow and impairs memory function in Alzheimer’s disease mouse models,” Nat. Neurosci. 22(3), 413–420 (2019). NANEFN 1097-6256 10.1038/s41593-018-0329-4 30742116 PMC6508667

[16] Fluri F. Schuhmann M. K. Kleinschnitz C., “Animal models of ischemic stroke and their application in clinical research,” Drug Des. Dev. Ther. 9, 3445–3454 (2015). 10.2147/DDDT.S56071 PMC4494187 26170628

[r17] Murphy T. H. et al., “Two-photon imaging of stroke onset in vivo reveals that NMDA-receptor independent ischemic depolarization is the major cause of rapid reversible damage to dendrites and spines,” J. Neurosci. 28(7), 1756–1772 (2008). JNRSDS 0270-6474 10.1523/JNEUROSCI.5128-07.2008 18272696 PMC6671530

[r18] Zeng L. et al., “MicroRNA-210 overexpression induces angiogenesis and neurogenesis in the normal adult mouse brain,” Gene Ther. 21(1), 37–43 (2014). GETHEC 0969-7128 10.1038/gt.2013.55 24152581

[r19] Shimamura M. et al., “Therapeutic effects of systemic administration of the novel RANKL-modified peptide, MHP1, for ischemic stroke in mice,” Biomed Res. Int. 2018, 1–8 (2018). 10.1155/2018/4637084 PMC6091369 30151382

[20] Hakon J. et al., “Multisensory stimulation improves functional recovery and resting-state functional connectivity in the mouse brain after stroke,” NeuroImage Clin. 17, 717–730 (2018). 10.1016/j.nicl.2017.11.022 29264113 PMC5726755

[21] Janssen B. J. A. et al., “Effects of anesthetics on systemic hemodynamics in mice,” Am. J. Physiol. Circ. Physiol. 287(4), H1618–H1624 (2004). 10.1152/ajpheart.01192.2003 15155266

[22] Masamoto K. Kanno I., “Anesthesia and the quantitative evaluation of neurovascular coupling,” J. Cereb. Blood Flow Metab. 32(7), 1233–1247 (2012). 10.1038/jcbfm.2012.50 22510601 PMC3390804

[23] Szkulmowski M. Tamborski S. Wojtkowski M., “Spectrometer calibration for spectroscopic Fourier domain optical coherence tomography,” Biomed. Opt. Express 7(12), 5042 (2016). BOEICL 2156-7085 10.1364/BOE.7.005042 28018723 PMC5175550

[24] Watson B. D. et al., “Induction of reproducible brain infarction by photochemically initiated thrombosis,” Ann. Neurol. 17(5), 497–504 (1985). 10.1002/ana.410170513 4004172

[25] Baran U. et al., “Tail artifact removal in OCT angiography images of rodent cortex,” J. Biophotonics 10(11), 1421–1429 (2017). 10.1002/jbio.201600194 27600882 PMC5340634

[26] Hall C. N. et al., “Capillary pericytes regulate cerebral blood flow in health and disease,” Nature 508(7494), 55–60 (2014). 10.1038/nature13165 24670647 PMC3976267

[r27] Itoh Y. Suzuki N., “Control of brain capillary blood flow,” J. Cereb. Blood Flow Metab. 32(7), 1167–1176 (2012). 10.1038/jcbfm.2012.5 22293984 PMC3390803

[28] Moeini M. et al., “Compromised microvascular oxygen delivery increases brain tissue vulnerability with age,” Sci. Rep. 8(1), 8219 (2018). SRCEC3 2045-2322 10.1038/s41598-018-26543-w 29844478 PMC5974237

[29] Lee J. et al., “Motion correction for phase-resolved dynamic optical coherence tomography imaging of rodent cerebral cortex,” Opt. Express 19(22), 21258–21270 (2011). OPEXFF 1094-4087 10.1364/OE.19.021258 22108978 PMC3386793

[30] Zhang A. et al., “Methods and algorithms for optical coherence tomography-based angiography: a review and comparison,” J. Biomed. Opt. 20(10), 100901 (2015). JBOPFO 1083-3668 10.1117/1.JBO.20.10.100901 26473588 PMC4881033

[31] Pries A. R. Secomb T. W. Gaehtgens P., “Relationship between structural and hemodynamic heterogeneity in microvascular networks,” Am. J. Physiol. 270(2 Pt. 2), H545–53 (1996). AJPHAP 0002-9513 10.1152/ajpheart.1996.270.2.H545 8779829

[32] Park C. S. Payne S. J., “Modelling the effects of cerebral microvasculature morphology on oxygen transport,” Med. Eng. Phys. 38(1), 41–47 (2016). MEPHEO 1350-4533 10.1016/j.medengphy.2015.09.004 26499366 PMC4751405

[33] Sato Y. et al., “Three-dimensional multi-scale line filter for segmentation and visualization of curvilinear structures in medical images,” Med. Image Anal. 2(2), 143–168 (1998). 10.1016/S1361-8415(98)80009-1 10646760

[34] Frangi A. F. et al., “Multiscale vessel enhancement filtering,” Lect. Notes Comput. Sci. 1496, 130–137 (1998). LNCSD9 0302-9743 10.1007/BFb0056181

[35] Rezakhaniha R. et al., “Experimental investigation of collagen waviness and orientation in the arterial adventitia using confocal laser scanning microscopy,” Biomech. Model. Mechanobiol. 11(3–4), 461–473 (2012). BMMICD 1617-7959 10.1007/s10237-011-0325-z 21744269

[36] Hadjistassou C. Bejan A. Ventikos Y., “Cerebral oxygenation and optimal vascular brain organization,” J. R. Soc. Interface 12(107), 20150245 (2015).1742-5689 10.1098/rsif.2015.0245 25972435 PMC4590512

[37] Gould I. G. et al., “The capillary bed offers the largest hemodynamic resistance to the cortical blood supply,” J. Cereb. Blood Flow Metab. 37(1), 52–68 (2017). 10.1177/0271678X16671146 27780904 PMC5363755

[38] Sakadžić S. et al., “Large arteriolar component of oxygen delivery implies a safe margin of oxygen supply to cerebral tissue,” Nat. Commun. 5(1), 5734 (2014). NCAOBW 2041-1723 10.1038/ncomms6734 25483924 PMC4260810

[39] Fan Y. et al., “Endothelial progenitor cell transplantation improves long-term stroke outcome in mice,” Ann. Neurol. 67(4), 488–497 (2010). 10.1002/ana.21919 20437584 PMC3026588

[40] Kojima T. et al., “Subventricular zone-derived neural progenitor cells migrate along a blood vessel scaffold toward the post-stroke striatum,” Stem Cells 28(3), 545–554 (2010). 10.1002/stem.306 20073084

[41] Perez-de-Puig I. et al., “Neutrophil recruitment to the brain in mouse and human ischemic stroke,” Acta Neuropathol. 129(2), 239–257 (2015). 10.1007/s00401-014-1381-0 25548073

[42] Jickling G. C. et al., “Targeting neutrophils in ischemic stroke: translational insights from experimental studies,” J. Cereb. Blood Flow Metab. 35(6), 888–901 (2015). 10.1038/jcbfm.2015.45 25806703 PMC4640255

